# Acinar Cell Proliferation Promoted by BMP2 in Injured Mouse Parotid Gland: BMP2 Promotes Cell Proliferation in Parotid Gland

**DOI:** 10.1155/2023/1765317

**Published:** 2023-03-31

**Authors:** Megumi Yokoyama, Osamu Katsumata-Kato, Junko Fujita-Yoshigaki

**Affiliations:** Department of Physiology, Nihon University School of Dentistry at Matsudo, Chiba, Japan

## Abstract

**Objective:**

To identify factors that affect salivary gland recovery, we investigated the expression and function of bone morphogenetic protein 2 (BMP2) in mice.

**Materials and Methods:**

Using a micro clip, mice parotid glands were removed 7 days after the ligation of the unilateral parotid excretory duct. Thereafter, they were weighed and stained with hematoxylin and eosin, and *BMP2* expression was examined via real-time reverse transcription-polymerase chain reaction. Primary cultures of parotid glands were prepared, and BMP2 protein was added to the culture medium for 48 hr to examine its effect on cell proliferation. E-cadherin and vimentin expression was examined using western blotting. Finally, immunohistochemical staining using an anti-Ki67 antibody was performed.

**Results:**

Duct-ligated parotid glands weighed less than those that were collected after sham surgery and showed acinar cell atrophy. They also showed higher *BMP2* expression than control glands. Primary-cultured parotid acinar cells supplemented with BMP2 showed higher proliferative potential than control cells. Furthermore, they showed E-cadherin, but not vimentin, expression, and their percentage of Ki67-positive cells were higher than that corresponding to the controls.

**Conclusions:**

Injury to salivary glands by excretory duct ligation increased *BMP2* expression, which may be involved in maintaining salivary gland function by inducing acinar cell proliferation.

## 1. Introduction

The salivary glands are important exocrine glands located around the oral cavity and consist of acini that produce saliva and ducts that carry the saliva into the oral cavity. Saliva plays a role in digestion, antibacterial and bactericidal actions, and food mass formation. Decreased salivary secretion can cause various disorders, including xerostomia, resulting in a decreased quality of life. We aimed to identify the factors that restore the function of the reduced salivary glands to improve xerostomia. Duct ligation, i.e., ligating the ducts of an organ, is a common technique used in the pancreas, salivary glands, and liver to injure tissue [1–3]. Duct ligation in the salivary glands causes gland atrophy, a loss of acinar cells via apoptosis, and results in decreased saliva production [4, 5]. However, it has been shown that after the ligation is removed, the salivary gland recovers, owing to an increase in the number of acinar cells [6]. We have previously reported that the expression of cytokeratin 5, a salivary gland stem cell marker, also increased after duct ligation [7]. Therefore, it is likely that recovery factors are expressed within the atrophic salivary glands during duct ligation in the salivary gland.

The pancreas is structurally similar to the salivary glands and plays a role in the exocrine system akin to the salivary glands. Although the pancreas has both endocrine and exocrine functions, the majority remain exocrine in nature. Rastellini et al. [8] reported that duct ligation in the pancreas significantly increased bone morphogenetic protein 2 (*BMP2*) mRNA expression. We hypothesized that if duct ligation in the salivary glands also induces the expression of *BMP2*, it could be a recovery factor for the dysfunctional salivary glands. BMP2 is a member of the transforming growth factor beta (TGF-*β*) superfamily and is known to be a potent inducer of bone and cartilage formation [9, 10]. In recent years, BMP2 has been detected in organs other than bone and cartilage, such as kidneys [11], lungs [12], and blood vessels [13], which suggests that BMP2 has a variety of physiological functions besides bone and cartilage formation. *BMP2* expression in salivary glands has been found to be predominantly tumor-induced [14–17], and no study to date has examined *BMP2* expression after duct ligation in salivary glands.

In this study, we hypothesized that *BMP2* expression is induced by duct ligation in the salivary glands, akin to that observed after duct ligation in the structurally similar pancreas. Further, we aimed to clarify the role of BMP2 after tissue injury to the salivary glands by duct ligation.

## 2. Materials and Methods

### 2.1. Animals

Twenty-four 7-week-old male C57BL6/J mice (21–23 g) were used for duct ligation, and 12 5-week-old male C57BL6/J mice were used to obtain primary cultured cells. These mice were purchased from Sankyo Labo Service Corporation, Inc. (Tokyo, Japan). Standard food and water were provided ad libitum. Mice were bred in a constant environment of 24°C room temperature, 65% humidity, and a 12 hr light/dark cycle. Experiments were carried out in accordance with the guidelines laid down by the National Institute of Health (NIH) in the USA regarding the care and use of animals for experimental procedures or by the European Communities Council Directive of November 24, 1986 (86/609/EEC) and in accordance with local laws and regulations. This experiment was performed at the Nihon University School of Dentistry at Matsudo, in accordance with the institutional and national guidelines for the care and use of experimental animals with the approval of the Experimental Animal Committee of the Nihon University School of Dentistry at Matsudo (approval no. AP13MD021-1).

### 2.2. Duct Ligation and Parotid Gland Weight

Mice were injected intraperitoneally with a mixture of medetomidine hydrochloride (0.3 mg/kg), midazolam (4 mg/kg), and butorphanol tartrate (5 mg/kg). Under anesthesia, the unilateral parotid excretory duct was ligated, according to a previously reported procedure [18]. Salivary glands recover when the ligation is released 7 days after the duct ligation [19, 20]. We previously reported that parotid gland morphology showed weak atrophy of acinar cells in some samples on day 3 of duct ligation, whereas most acinar cells in the parotid gland were atrophied on day 7 of duct ligation [18]. Therefore, the period of duct ligation was set at 7 days for the present study. The mice were weighed on day 7 after ligation, and the parotid glands were excised [18] and weighed immediately. Parotid gland weight was divided by body weight to calculate parotid gland weight per body weight. Mice used as controls (*n* = 10) underwent sham operations and were maintained for the same period as the experimental group. In brief, a unilateral cheek was incised and sutured.

### 2.3. Hematoxylin and Eosin (H&E) Staining

The extracted parotid glands were fixed in 10% formalin diluted in phosphate-buffered saline. They were then embedded in paraffin and cut into 3 *µ*m-thick sections. Tissue sections were stained with H&E and examined under a microscope (Olympus BX51; Olympus, Tokyo, Japan). Images were acquired using an Olympus CP12 system.

### 2.4. Real-Time Reverse Transcription-Polymerase Chain Reaction (RT-PCR)

TRIzol reagent (Thermo Fisher Scientific, Waltham, MA, USA) and the RNeasy Mini Kit (Qiagen, Hilden, Germany) were used to isolate total RNA according to the manufacturer's instructions. The optical density was measured at 260 nm to determine the quantity of the isolated RNA. The mRNA expression level was determined using the One Step TB Green PrimeScript PLUS RT-PCR Kit (Takara Bio, Shiga, Japan) and a Thermal Cycler Dice (Takara Bio). Primer pairs used for the amplification of *BMP2* were 5′-TGTGGAGACTCTCTCAATGGAC-3′ (forward) and 5′-GGAAGCAGCAACACTAGAAGAC-3′ (reverse); those used for the amplification of glyceraldehyde 3-phosphate dehydrogenase (*GAPDH*) were 5′-CTGGAGAAACCTGCCAAGTATG-3′ (forward) and 5′-CAACCTGGTCCTCAGTGTAG-3′ (reverse). Relative mRNA expression levels were calculated using the *ΔΔ*Ct method. To obtain relative RNA equivalents for each sample, *GAPDH* levels were normalized. Each sample was analyzed in duplicate to confirm the reproducibility of the findings.

### 2.5. Primary Culture of Parotid Acinar Cells

Five-week-old mice were anesthetized with an intraperitoneal injection of a mixture of medetomidine hydrochloride (0.3 mg/kg), midazolam (4 mg/kg), and butorphanol tartrate (5 mg/kg), and the parotid glands were extracted. Acinar cells were isolated from the parotid glands as previously described [21]. Isolated acinar cells were diluted with Waymouth's medium containing 10% rat serum, 1% ITS-X supplement, 1% penicillin–streptomycin, 1 *µ*m hydrocortisone, and 10 nM cystatin, and cultured in collagen I-coated dishes (Iwaki, Tokyo, Japan) at 37°C in a 5% CO_2_ incubator. One day after cellular isolation, the cells were then divided into groups and cultured as follows: the first group had the same Waymouth's medium concentration at the initial culture medium (10% rat serum), the second had Waymouth's medium concentration adjusted to 2% rat serum, and the third had Waymouth medium supplemented with 100 ng/mL recombinant human BMP2 (Chicago, Humanzyme, IL, USA) to 2% rat serum. The cells were seeded at a concentration of 0.02–0.06 mg/mL cells per well in a 96-well plate for the cell proliferation assay.

### 2.6. Cell Proliferation Assay

The cellular proliferation capacity at 48 hr after the addition of BMP2 was determined using the Counting Kit-8 (Dojindo Laboratories, Kumamoto, Japan) according to the manufacturer's protocol.

### 2.7. Sodium Dodecyl Sulphate–Polyacrylamide Gel Electrophoresis (SDS-PAGE) and Western Blotting

After 48 hr of incubation, the cells were lysed in 20 mM HEPES (pH 7.4) containing 0.1% Triton X-100 and 1× complete protease inhibitor cocktail, 25x (Roche Diagnostics, Basel, Switzerland). Equal amounts of protein were separated by SDS–PAGE and transferred to a Hybond-LFP 0.2 polyvinylidene difluoride membrane (GE Healthcare, Buckinghamshire, UK). The membrane was blocked in ECL Blocking Agent (GE Healthcare) and subjected to immunoblotting with antibodies against E-cadherin (Cell Signaling Technology, Danvers, MA, USA), vimentin (Cell Signaling Technology), and *β*-actin (Abcam, Cambridge, UK). NIH3T3 whole cell lysate (Novus Biologicals, Centennial, CO, USA) was used as a positive control for mesenchymal markers. The ChemiDoc MP imaging system (Bio-Rad, Hercules, CA, USA) was used to obtain images, and fluorescence intensities were quantified using the ImageLab software (Bio-Rad).

### 2.8. Anti-Ki67 Immunohistochemical Staining and Positive Cell Rate

The excised parotid glands were immediately fixed in a 10% formalin-neutral buffer solution (Wako Pure Chemical Industries, Ltd. Osaka, Japan) and paraffin-embedded using conventional methods. The paraffin block was sectioned into 3 *µ*m-thick sections, which were mounted on glass slides. Ki67 staining and counting of the number of positive cells were performed according to a previously reported procedure [18]. The positive cell rate was determined by dividing the number of positive cells by the total number of cells.

### 2.9. Statistical Analysis

All values are reported as mean ± standard deviation (SD). All experimental data were analyzed using Student's *t*-test. Statistical significance was set at *P* < 0.05.

## 3. Results

### 3.1. Duct Ligation Induces Atrophy and BMP2 Expression in Parotid Glands

**Figure 1:**
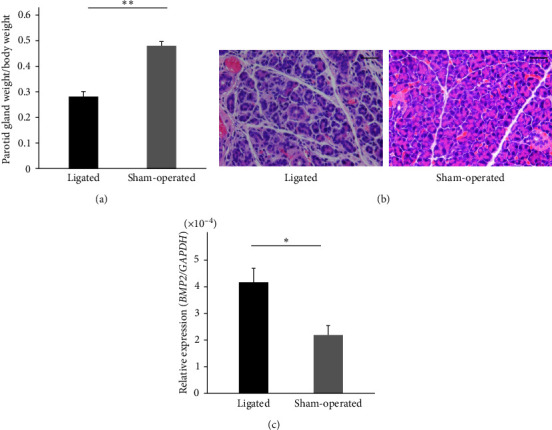
Changes in the parotid gland and expression of BMP2 after 7 days of unilateral parotid duct ligation in mice. (a) Weight of duct-ligated and sham-operated parotid glands. The duct-ligated parotid gland showed a significant decrease in weight.  ^*∗∗*^Indicates significance at *P* < 0.01. Data were shown as mean ± SD (*n* = 5). (b) Hematoxylin and eosin (H&E) staining of parotid glands in the ligated and sham-operated glands. Scale bar = 30 *µ*m. (c) Changes in BMP2 mRNA expression in duct-ligated and sham-operated parotid glands. BMP2 mRNA expression increased significantly in the duct-ligated parotid gland.  ^*∗*^Indicates significance at *P* < 0.05. Data were shown as mean ± SD (*n* = 5).

Weight measurements and histological examinations were performed to confirm the effects of duct ligation on the parotid gland. On day 7, after duct ligation, the average weight of the parotid gland was approximately half that of the sham-operated parotid gland (Figure 1(a)). Macroscopic parotid gland size was also smaller in the duct-ligated parotid glands (data not shown) than that of the controls. Morphologically, the parotid gland with a duct ligation for 7 days showed atrophic acinar cells compared to the normal acinar cells in the parotid gland with the sham-operation (Figure 1(b)). Duct-like structures were observed in some of the atrophied acinar cells due to duct ligation, and the connective tissue between the lobules and the spaces between the acinar and duct cells were littered with prominent mononuclei-containing cells (Figure 1(b)). Normal histology was observed in the sham-operated parotid glands (Figure 1(b)).

Next, we examined the expression level of *BMP2* in duct-ligated parotid glands to confirm whether *BMP2* was expressed after duct ligation of the parotid gland. The expression level of *BMP2* was significantly higher in the duct-ligated parotid gland than in the sham-operated gland (Figure 1(c)). These experiments indicate that duct ligation of the parotid gland results in the gland's atrophy owing to a reduction in the size of the glandular cells and an increase in the expression of *BMP2*.

### 3.2. BMP2 Affects the Proliferative Potential of Primary Cultures of Parotid Acinar Cells

As mentioned earlier, we predicted that the recovery factors of acinar cells would be expressed during the period of duct ligation. As *BMP2* expression was increased in duct-ligated parotid glands, the effect of BMP2 on cell proliferation was subsequently investigated using in vitro primary cultures of parotid acinar cells.

**Figure 2:**
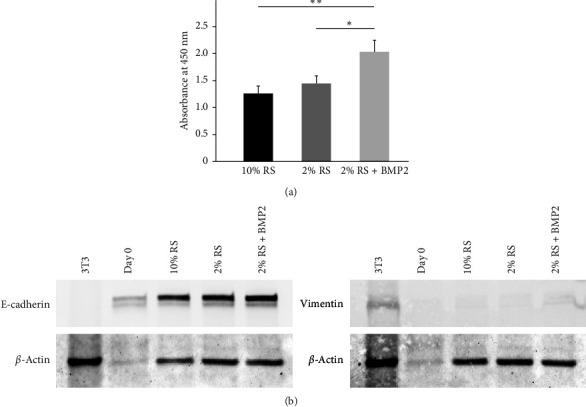
Mouse parotid acinar cells in primary culture. (a) Cell proliferative capacity of BMP2-added (100 ng/mL) and nonadded (control) groups after 48 hr of culture. The proliferative capacity of BMP2-added cultured cells was significantly increased compared to that in the control group.  ^*∗∗*^Indicates significance at *P* < 0.01,  ^*∗*^*P* < 0.05. Data were shown as mean ± SD. Three independent experiments were performed. (b) Protein expression of E-cadherin (an epithelial marker) and vimentin (a mesenchymal marker) in cultured cells of each group at 48 hr after the addition of BMP2. NIH3T3 (3T3) was used as a positive control for mesenchymal markers. RS: rat serum.

The proliferative capacity of the primary cultured cells 48 hr after the addition of BMP2 was significantly higher than that of the cells in the 2% and 10% rat serum control groups without BMP2 (Figure 2(a)). Parotid gland acinar cells are epithelial-derived cells. Therefore, to confirm that the proliferated cells were epithelial cells and not fibroblasts, we investigated the protein expression of E-cadherin, an epithelial marker, and vimentin, a mesenchymal marker, to clarify whether the cells retained their epithelial functions after 48 hr of culture. E-cadherin was detected in all cultured cells with or without BMP2 (Figure 2(b)). Meanwhile, vimentin was not detected in any cells except in the positive control (Figure 2(b)).

### 3.3. Increased Cell Proliferative Capacity in Duct-Ligated Parotid Glands

**Figure 3:**
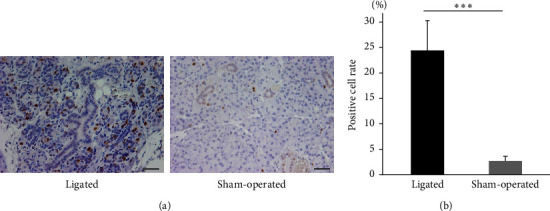
Cell proliferative capacity of parotid glands 7 days postduct ligation. (a) Immunohistochemical staining of Ki67 (brown) in ligated parotid and sham-operated glands. Scale bar = 30 *µ*m. (b) Ki67-positive cell rate of ligated and sham-operated glands. The cell proliferation rate increased significantly in duct-ligated parotid glands. The number of cells was counted in three fields (magnification 400×) of each sample.  ^*∗∗∗*^Indicates significance at *P* < 0.005. Data were shown as mean ± SD (*n* = 5).

To confirm the in vitro findings, the proliferative capacity of the parotid tissue was evaluated in vivo by using Ki67, a cell proliferation marker. Ki67-positive cells were more conspicuous in duct-ligated parotid glands than in the sham-operated parotid glands (Figure 3(a)). Ki67 were mainly detected in the epithelial structures, although duct ligation increased the area of mesenchyme. In addition, Ki67-positive cells were rarely seen in large ducts, which may be excretory ducts. However, confident identification of cell types such as acinar, duct, and mesenchymal cells is difficult because of large histological changes. Thus, we counted the number of Ki67-positive cells without discrimination of cell types. The Ki67-positive cell rate was 24.6% in the duct-ligated parotid glands and 2.8% in the sham-operated parotid glands, with a significantly increased Ki67-positive cell rate in the duct-ligated parotid glands (Figure 3(b)). Previous studies also showed that after 7-day duct ligation, histological staining showed the loss of acinar cells and prominence of ducts. However, Aure et al. [22] reported that acinar cells changed to duct-like and survived in ligated glands. Removal of ligation caused the recovery of acinar cells by proliferation and expansion of the survived acinar-derived cells [22]. Thus, the Ki67-positive cells in the epithelial structures may contribute to the recovery of acinar cells.

## 4. Discussion

The purpose of this study was to clarify whether *BMP2* expression is induced by the ligation of the salivary gland excretory duct, to identify salivary gland recovery factors, and to investigate the role of BMP2. To date, there have been no reports describing the relationship between salivary glands and BMP2. In this study, *BMP2* expression was induced by duct ligation of the parotid gland, and the proliferative ability was promoted by the addition of BMP2 in the primary cultured cells of the parotid gland. In the in vivo experiment, the proliferative ability increased in the duct-ligated parotid gland compared with that of the controls.

Histological images of the parotid gland that were taken 7 days after ligation of the unilateral parotid excretory duct showed atrophy and reduction of acinar cells, increased duct-like structures, and an increased number of cells with prominent mononuclei in the intercellular spaces (Figures 1(a) and 1(b)). These findings are similar to those reported in previous studies [19, 20]. Ikai et al. [19] reported that the mononuclear cells that appear in duct ligations were an inflammatory cellular infiltrate consisting mainly of neutrophils and macrophages. It has also been reported that ligation of ducts in organs with exocrine gland functions, such as the liver [20], pancreas [21], and lacrimal gland [23], induces inflammation; therefore, it can be inferred that duct ligation in salivary glands also causes inflammation. The salivary glands are important organs that secrete saliva, and their inflammation causes a decrease in function [24, 25]. Although the parotid tissue was atrophic on day 7 of duct ligation, the gene expression of *BMP2* was higher than that in controls (Figure 1(c)). Recent studies have reported the expression of *BMP2* in various organs, such as the pancreas [8, 26], blood vessels [13, 27], kidney [11, 28], heart [29], and periodontal ligament [30]. Although there have been previous reports of *BMP2* expression in salivary glands in adenocarcinoma cell lines [31] and mixed tumors [17], this is the first study to report that *BMP2* expression is induced by duct ligation.

Using parotid gland primary-cultured acinar cells, we examined the effect of *BMP2* on cell proliferation and demonstrated that *BMP2* promoted the proliferation of acinar cells (Figure 2(a)). These salivary glands were capable of regeneration. When salivary glands undergo duct ligation, the acinar cells atrophy and lose their function, but when duct ligation is released, the atrophied acinar cells recover to their original state [32]. Previous studies have reported that *Δ*Np63, a p63 isoform [19], and epiregulin [33], which show high expression after duct ligation compared to nonduct ligation, are factors involved in regeneration and recovery. Thus, *BMP2* is involved in regeneration and recovery.

BMP2 is a member of the TGF-*β* superfamily. TGF-*β* is known to be involved in the pathogenesis of fibrosis in various organs. Interestingly, BMP2, in contrast to TGF-*β*, has antifibrotic effects in the kidney [34], lung [12], liver [35], and pancreas [8]. TGF-*β* is also expressed in salivary glands involved in fibrosis caused by duct ligation [36]. Therefore, *BMP2* induced by duct ligation may counteract fibrosis of salivary glands while promoting cell proliferation to preserve salivary gland function after duct ligation injury.

Duct ligation in salivary glands is often used as a model for salivary gland injury. In in vivo experiments, the number of Ki67-positive cells increased on day 7 after duct ligation, indicating that injury by duct ligation promoted cell proliferation (Figures 3(a) and 3(b)). In previous studies, Walker and Gobé [5] and Takahashi et al. [37] reported that ligation did not increase proliferative capacity. They evaluated cell proliferation in thymidine index and BrdU. Thymidine index detects the G1 and early S phase of the cell cycle. BrdU evaluates the S phases of the cell cycle. Here, the entire cell cycle (G1, S, G2, and M phases) was evaluated using Ki67 antibody. Therefore, it is considered that different results from the previous studies were obtained because cycles other than the S phase of cell proliferation were also evaluated. Another proliferation marker, proliferating cell nuclear antigen, has been reported to be increased by duct ligation [6], which suggests that duct ligation in salivary glands triggers the cell proliferation cycle. As duct ligation in salivary glands induces both *BMP2* expression and cell proliferation, it is suggested that *BMP2*, which shows high expression after duct ligation, affects cell proliferation in the salivary glands.

Dry mouth or xerostomia is a condition wherein the oral cavity becomes excessively dry because of decreased saliva production. Xerostomia is caused by either systemic diseases, neurological disorders, medicines, or disorders of the salivary glands. Furthermore, irradiation during treatment of the head and neck destroys saliva-producing acinar cells and leads to functional damage that decreases saliva secretion [38, 39]. A decrease in saliva production leads to mucosal inflammation, reduced taste sensitivity, and difficulty eating and swallowing, which, in turn, worsens the quality of life. If untreated, xerostomia can lead to nutritional deficiencies, worsening of mood, and depression [40]. In recent years, research has been conducted on a radical treatment that involves culturing salivary gland primordium ex vivo and then transplanting these salivary glands [41, 42]. However, the current treatment mainly involves symptomatic therapy using drugs and artificial saliva, but a treatment method to directly restore salivary gland function has not yet been established. Therefore, we hypothesized that BMP2 would promote the proliferation of glandular cells and that the increase in glandular cell numbers could ameliorate the decline in salivary function.

Although further detailed investigations on the role of BMP2 in salivary glands are required, this study provides the first evidence that BMP2 in salivary glands promotes an increase in the number of glandular acinar cells, which may help to improve xerostomia.

## Data Availability

The data that support the findings of this study are available in this article.
